# Leveraging machine learning for duration of surgery prediction in knee and hip arthroplasty – a development and validation study

**DOI:** 10.1186/s12911-025-02927-7

**Published:** 2025-03-03

**Authors:** Benedikt Langenberger, Daniel Schrednitzki, Andreas Halder, Reinhard Busse, Christoph Pross

**Affiliations:** 1 Department of Health Care Management, Technische Universität Berlin, Berlin, Germany; 2 Chair of Digital Health, Economics & Policy, Hasso-Plattner-Institute, Potsdam, Germany; 3 Department of Orthopaedic, Trauma, Hand and Reconstructive Surgery, Sana Klinikum Lichtenberg, Berlin, Germany; 4 Department of Orthopedic Surgery, Sana Klinken Sommerfeld, Brandenburg, Germany

**Keywords:** Duration of surgery, Machine learning, Patient-reported outcome measures

## Abstract

**Background:**

Duration of surgery (DOS) varies substantially for patients with hip and knee arthroplasty (HA/KA) and is a major risk factor for adverse events. We therefore aimed (1) to identify whether machine learning can predict DOS in HA/KA patients using retrospective data available before surgery with reasonable performance, (2) to compare whether machine learning is able to outperform multivariable regression in predictive performance and (3) to identify the most important predictor variables for DOS both in a multi- and single-hospital context.

**Methods:**

eXtreme Gradient Boosting (XGBoost) and multivariable linear regression were used for predictions. Both models were applied to both the whole dataset which included multiple hospitals (3,704 patients), and a single-hospital dataset (1,815 patients) of the hospital with the highest case-volumes of our sample. Data was split into training (75%) and test data (25%) for both datasets. Models were trained using 5-fold cross-validation (CV) on the training datasets and applied to test data for performance comparison.

**Results:**

On test data in the multi-hospital setting, the mean absolute error (MAE) was 12.13 min (HA) / 13.61 min (KA) for XGBoost. In the single-hospital analysis, performance on test data was MAE 10.87 min (HA) / MAE 12.53 min (KA) for XGBoost. Predictive ability of XGBoost was tended to be better than of regression in all setting, however not statistically significantly. Important predictors for XGBoost were physician experience, age, body mass index, patient reported outcome measures and, for the multi-hospital analysis, the hospital.

**Conclusion:**

Machine learning can predict DOS in both a multi-hospital and single-hospital setting with reasonable performance. Performance between regression and machine learning differed slightly, however insignificantly, while larger datasets may improve predictive performance. The study found that hospital indicators matter in the multi-hospital setting despite controlling for various variables, highlighting potential quality differences between hospitals.

**Trial registration:**

The study was registered at the German Clinical Trials Register (DRKS) under DRKS00019916.

**Supplementary Information:**

The online version contains supplementary material available at 10.1186/s12911-025-02927-7.

## Highlights

Machine learning predicts surgery duration for hip and knee arthroplasty with a mean error of slightly above 10 min.

Performance was comparable for a multi-hospital and a single-hospital setting.

Hospital indicators were highly relevant predictors in the multi-hospital setting.

Patients age, body composition and surgery-team related variables were major drivers of predictive power in both settings.

**Supplementary Information:**

The online version contains supplementary material available at 10.1186/s12911-025-02927-7.

## Background

Extended duration of surgery (DOS) for hip or knee arthroplasty (HA/KA) patients has been associated with adverse outcomes such as increased risk of revision [1], readmission, minor or major complications [2], surgical site infection [3], periprosthetic infection [4], deep infection [5] and renal impairment [6]. Identification of patients at risk of high DOS through accurate DOS prediction would support medical decision-making. For patients with high predicted DOS, countermeasures to reduce DOS may be introduced to mitigate abovementioned risks. In addition to patient safety, accurate DOS prediction can increase hospital operating room (OR) scheduling efficiency. When patients were previously known to have low DOS, schedules can be tightened, and throughput increased. When patients were known to have high DOS, schedules can be adapted, waiting times reduced, and shifts of planned surgeries prohibited.

Yet data-driven DOS planning is rarely performed in orthopedic clinics. However, advanced analytics could support DOS prediction and improve OR optimization. Recently, machine learning (ML), a subbranch of artificial intelligence (AI), has been successfully applied in the field of knee and hip arthroplasty for binary [7–9], multiclass [10–12], or regression prediction tasks [9, 13, 14]. Specifically supervised ML, a set of methods that learn from data where the outcome is labelled (i.e. the values of the outcomes are known during model training) [15], could be applied in such a task. ML differs from classical statistical methods such as regression in that it can handle very high-dimensional data, variable selection, complex interactions and non-linear relationships between variables without human specification [16].

Two recently published studies from Germany aimed to develop ML models for pre-operative binary prediction of DOS for total knee arthroplasty (TKA) or total hip arthroplasty (THA) patients [17, 18]. Both studies predicted whether patients were at risk of ‘irregular surgery time’ (longer or shorter than normal) [17]. Although the studies reported high predictive performance of the models, it remained unclear whether a patient was at risk of low or high DOS due to the authors definition of ‘irregular surgery time’. Yeo et al. [19] predicted TKA patients at risk of long duration of surgery time with good performance, however did not describe how exactly long duration was defined. Although binary prediction studies are helpful in detecting patients at risk of, e.g., high DOS, they are of limited practical relevance with respect to OR scheduling. Therefore, studies with DOS as continuous outcome are of more practical relevance [20].

That said, this study aimed to overcome shortcomings of previous research. We applied ML to perform a continuous outcome prediction (i.e. “regression task” [21]) for DOS. Here, we intend to perform a proof-of-concept study to test how prediction models can perform and ascertain whether practical testing and potential implementation may be useful. Because such an application for DOS prediction can be run for single-hospital settings, but also for providers that operate multiple hospitals (both private as in Germany, but also public as e.g. the National Health Service in England), we demonstrate the performance for both single- and multi-hospital settings. As part of the multi-hospital setting, we show how individual hospitals influence predictions, demonstrating how hospital operators may use such an approach also to benchmark their hospitals for quality or efficiency improvements.

To make predictions, we trained an eXtreme Gradient Boosting (XGBoost), a state-of-the art (ML) algorithm [22] that performed well in previous research [17, 18] and tested it on unseen test data (development and validation study). We compare the performance of all XGBoost models to linear multivariable regression models. Our results may help to optimize OR utilization by predicting DOS, potentially supporting OR resource planning decisions and enhancing patient safety by identifying patients at risk of long surgery.

## Data and methods

### Data source and missing data

We used data from the PROMoting Quality study, a randomized controlled trial which is described in detail in Kuklinski et al. [23]. The study was approved by the Charité’s Ethics Committee, Berlin (EA4/169/19). Further responsible ethical review committees of participating hospitals (Medical Chamber Hamburg, Medical Chamber Schleswig-Holstein, Hannover Medical School, Friedrich-Schiller University Jena, and Medical Chamber Brandenburg) agreed with the decision. The study was designed as a multi-centered, single-blinded randomized controlled trial that included nine German hospitals. The initial aim of the PROMoting Quality study was to identify whether patients that used post-discharge patient-reported outcome measures and received an alert when they are on an unfavorable trajectory improve outcomes of these patients one year after surgery, and therefore, sample size determination and power were derived (see Kuklinski et al.). All patients that underwent KA or HA at the respective clinics from 2019 to 2021 were eligible for the study. Patients were excluded if they were younger than 18, came through an emergency admission, had a tumor endoprosthesis, osteopathic neoplasm(s) or revision. As the RCT intervention started after hospital discharge, we were able to include both intervention and control group patients in this paper, since peri-operative data were unaffected by the intervention. Hence, this paper reflects a secondary analysis of the data generated in the PROMoting Quality study.

**Fig. 1:**
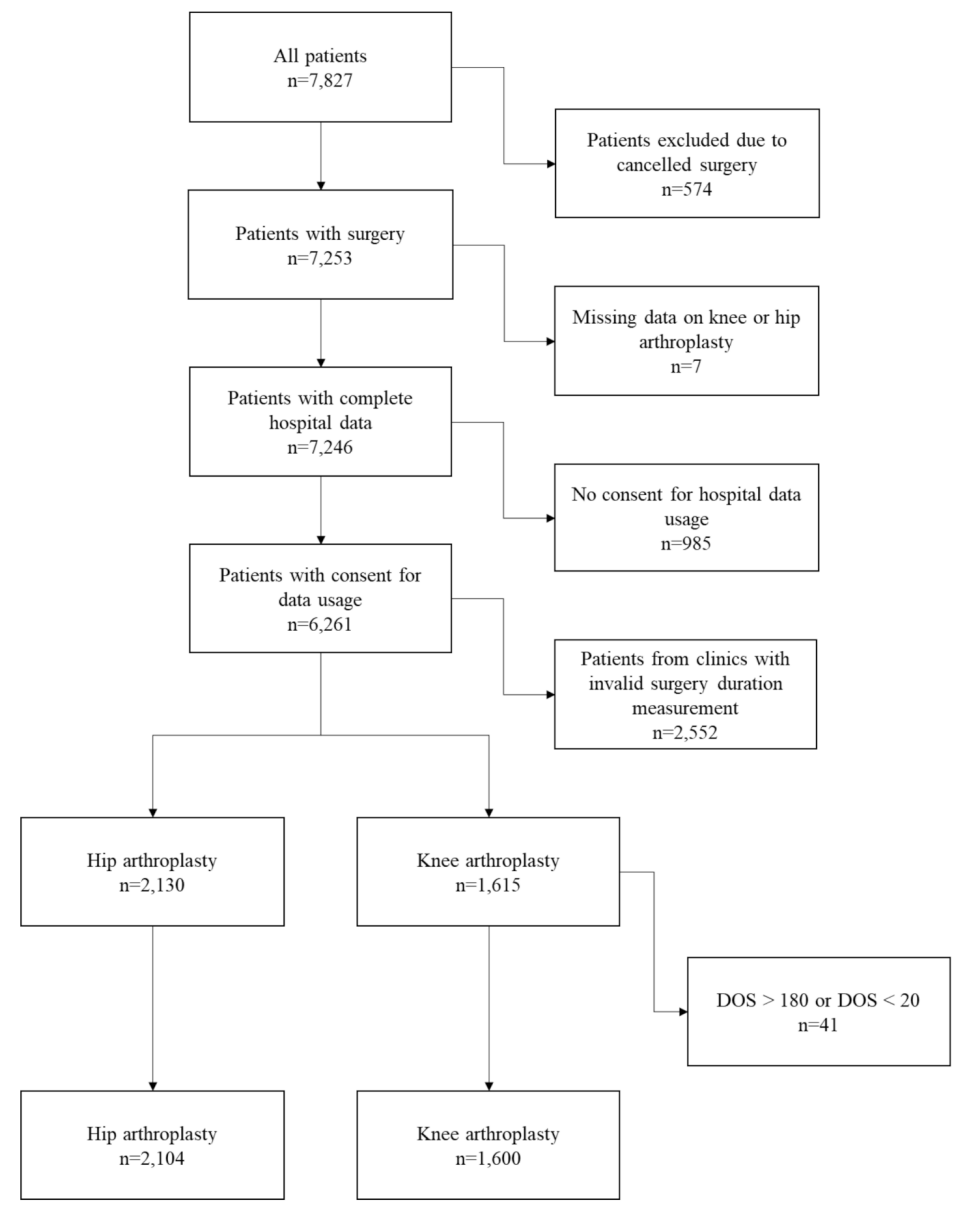
Patient selection flowchart

Originally, the dataset included 7,827 hip and knee arthroplasty patients. After removing observations of patients with cancelled surgery (*n* = 574), missing data on procedure (*n* = 7) and missing consent for hospital data usage (*n* = 985), the sample included 6,261 patients. Of those, 3,411 patients underwent HA and 2,850 patients underwent KA. We found that in two hospitals, data on DOS was not well distributed, with certain values (“60” and “90” min. in hospital 4; only 10-min. distances in hospital 9) occurring in unnatural frequency due to unreliable reporting of DOS by the two clinics (see Supplementary File 1). Therefore, we excluded these hospitals from further analysis, leaving 2,104 HA and 1,600 KA patients in our final sample (Fig. 1).

Missing data of any type were imputed with random forest imputation using the missForest package in R [24]. After assessment of medical professionals, we excluded extreme outliers of DOS (i.e. DOS > 180; DOS < 20) since their DOS values were assumed to be unreasonable (and extremely rare).

### Input variables

As predictors, we included all variables available in our dataset that we assume to be known prior to surgery at the time of model application in practice, namely more than 230 variables (see Supplementary File 2). These comprised sociodemographics, surgical history of the joint, prior diagnosis of arthrosis, previous therapy of the joint, behavioral variables (e.g. smoking), baseline PROM scores (PROMIS depression, PROMIS fatigue, EQ-5D-5 L, EQ-VAS, HOOS-PS/KOOS-PS), surgery team related variables (e.g. number of specialists (i.e., surgeons who have completed their residency training in the specific field) or number of surgeries performed by the surgeon) and dummy variables indicating the hospital to control for hospital effects on DOS (only in the multi-hospital analysis). All predictors of categorial nature were transformed into dummies via one-hot encoding.

### Study sample characteristics

Of the 3,709 included patients, 57% underwent HA, and 43% underwent KA. On average, patients in both indications were relatively similar with respect to age (HA: 66.3 years; KA: 67.0 years), gender (HA: 46% male; KA: 47% male) and Charlson weighted comorbidity score (HA: 0.42; KA: 0.42). Body mass index (BMI) was slightly higher for KA patients (BMI = 30.6 kg/m²) compared to HA patients (BMI = 28.0 kg/m²), as also indicated by higher weight (HA: 83.4 vs. KA: 90.9 kg) despite having the same average height (172 centimeters). Pre-operative PROM scores were slightly better for KA than for HA patients. KA patients had higher EQ-VAS scores (HA: 56.0; KA: 57.8) and higher EQ-5D-5 L scores (HA: 0.59; KA: 0.62) compared to HA patients. Mean HOOS-PS was 47.3 (HA), and mean KOOS-PS was 42.5 (KA). DOS was shorter for HA (55.9 min) than for KA patients (62.0 min) (Table 1).

**Table 1:** Characteristics of the study population grouped by indication

	Hip arthroplasty (*N* = 2,104)	Knee arthroplasty (*N* = 1,600)
**Age (years)**		
Mean (SD)	66.3 (10.4)	67.0 (9.0)
Median [Min, Max]	67.0 [20.0, 93.0]	67.0 [35.0, 87.0]
**Male**		
Mean (SD)	0.46 (0.50)	0.47 (0.50)
Median [Min, Max]	0 [0, 1.00]	0 [0, 1.00]
**Body mass index**		
Mean (SD)	28.0 (5.6)	30.6 (5.9)
Median [Min, Max]	27.0 [16.5, 114.0]	29.5 [8.0, 64.9]
**Weight**		
Mean (SD)	83.4 (18.6)	90.9 (19.5)
Median [Min, Max]	81.0 [45.0, 193]	89.0 [45.0, 176]
**Height**		
Mean (SD)	172 (9.8)	172 (10.2)
Median [Min, Max]	172 [81, 200]	172 [146, 250]
**Charlson weighted score**		
Mean (SD)	0.42 (0.82)	0.42 (0.76)
Median [Min, Max]	0 [0, 6.0]	0 [0, 5.0]
**EQ-5D-5 L**		
Mean (SD)	0.59 (0.26)	0.62 (0.25)
Median [Min, Max]	0.67 [-0.46, 1.00]	0.72 [-0.29, 1.00]
**EQ-VAS**		
Mean (SD)	56.0 (19.8)	57.8 (19.4)
Median [Min, Max]	57.5 [0, 100]	60.0 [0, 100]
**HOOS-PS/KOOS-PS**		
Mean (SD)	47.3 (16.0)	42.5 (12.1)
Median [Min, Max]	46.1 [0, 100]	40.3 [5.6, 100]
**DOS (mins)**		
Mean (SD)	55.9 (21.7)	62.0 (20.5)
Median [Min, Max]	51.0 [24, 180]	58.0 [20, 180]

^1^As especially many patients had DOS of 90 min (upper quantile), the high DOS group was substantially smaller than the low DOS group

### Outcome definition

As outcome, we used DOS in minutes. DOS was reported from all clinics using routinely available surgery time collection data of the hospitals enterprise resource planning systems. Specifically, DOS was reported as surgeon-controlled-time, which is the difference between “close time” (i.e. incisions made during surgery have been closed) and “surgical incision time” (i.e. time of first surgical cut) [25].

### Predictive modeling

As machine learning prediction methods, we applied XGBoost and linear multivariable regression. XGBoost is a tree-based ML algorithm and a further development of the original gradient boosting developed by Friedmann 2001 [26]. This machine learning method is open-source and known for its typically very high performance. XGBoost is designed for speed and performance, incorporating several innovations such as a novel tree learning algorithm for handling sparse data, weighted quantile sketch for approximate tree learning, and a block structure for parallel learning. These optimizations make XGBoost not only robust and accurate but also extremely fast, often outperforming other gradient boosting methods by an order of magnitude in terms of computational speed [22]. Linear multivariable regression was used as a non-ML baseline comparison method to compare whether an advanced ML algorithm such as XGBoost is able to outperform a traditional regression method.

We used the TRIPOD Checklist: Prediction Model Development in order to ensure high-quality prediction model development.

### Data split and hyperparameter selection

The dataset in each analysis (multi- and single-hospital) was split into 75% training and 25% test data. Hyperparameter tuning was performed on the training dataset using 5-fold cross-validation (CV) [27] and random search [28] to optimize the XGBoost model for the given problem. The fine-tuned XGBoost model was then applied to the test dataset for performance assessment on unforeseen data. Linear multivariable regression performance was also reported on the training dataset using CV and on unforeseen test data.

All analyses were conducted using R version 4.0.0 (R Foundation for Statistical Computing) and RStudio. For the XGBoost algorithm, the package ‘xgboost’ was used. For linear multivariable regression, the basic ‘lm’ function in R was applied.

### Performance measurement

Performance was evaluated on training (with CV) and test data for both the full sample analysis and the single hospital deep dive using the same set of performance measures. As a result of predicted and observed outcome, we were able to compute several metrics commonly applied in prediction tasks for continuous outcomes. As main metric, we used the mean absolute error (MAE). The MAE is derived as

1 $$\:MAE = \frac{1}{n}\sum\nolimits_{i = 1}^n {\left| {ob{s_i} - pre{d_i}} \right|},$$

where $\:{obs}_{i}$ is the observed outcome value, and $\:{pred}_{i}\:$is the predicted outcome by the respective model. We further report the root mean squared error (RMSE), which is derived as

2 $$\:RMSE = \:\sqrt {\frac{1}{n}\sum\nolimits_{i = 1}^n {{{(ob{s_i} - pre{d_i})}^2}} }.$$

The RMSE is commonly reported in prediction studies with continuous outcomes [29]. As a measure of percentage deviation of predictions from the actual values, we report mean absolute percentage error (MAPE), which is formally defined as

3 $$MAPE{\text{ }} = \:\frac{1}{n}\sum\nolimits_{i = 1}^n {\left| {\frac{{ob{s_i} - pre{d_i}}}{{ob{s_i}}}} \right|}.$$

The MAPE acts as a relative performance indicator, so that the performance of our models can be interpreted with respect to average DOS, and understand the deviation in terms of percentage.

### Feature importance

Feature importance was derived using SHapley Additive exPlanation (SHAP) analysis. SHAP analysis is a game-theory-based approach that illustrates how predictor variables influence predictions in ML studies [30]. SHAP analysis is therefore a method to overcome the “black-box” problem of AI and a step towards explainable AI. SHAP decomposes individual prediction differences from the expected value additive into features contributions [31].

## Results

**Fig. 2:**
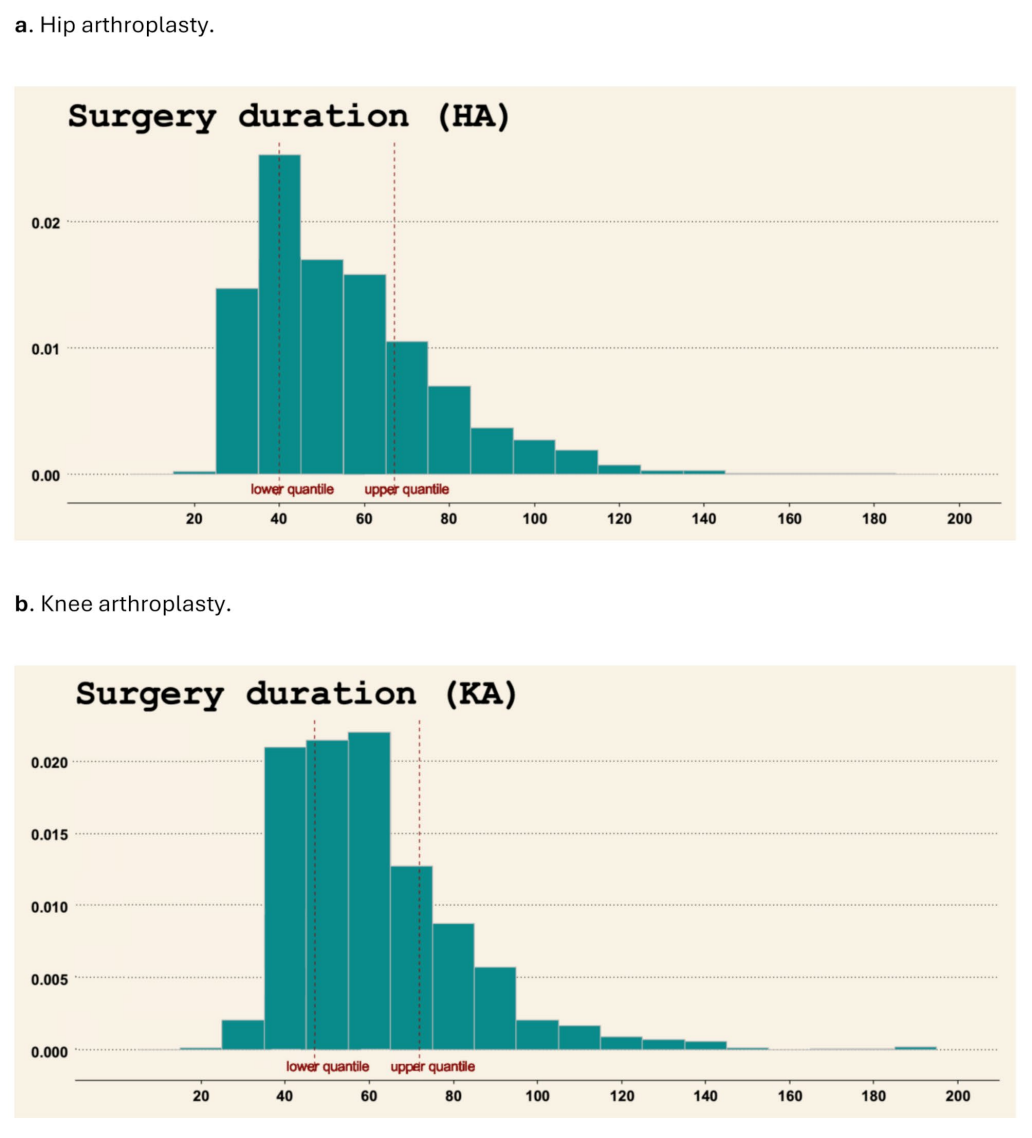
Distribution of DOS in hip and knee arthroplasty patients

The distribution ofDOS was skewed to the left for both KA and HA patients (Fig. 2). For HA patients, the lower quantile was 40 min and the upper quantile 67 min. For KA patients, the lower quantile was 47 min and upper quantile 72 min.

### Multi-hospital analysis

#### Performance on training data (multi-hospital analysis)

The XGBoost model with the selected hyperparameters achieved a cross-validated performance of MAE 12.60 min (12.10–13.11 min), RMSE 17.53 min (16.64–18.46 min), and MAPE 22.55% (21.6–23.52%) in the HA sample. In the KA sample, it achieved MAE 13.21 min (12.64–13.79 min), RMSE 18.04 min (17.11–19.00 min), and MAPE 20.94% (20.07–21.84%).

Linear multivariable regression performed slightly worse, yielding an MAE of 13.26 min (12.72–13.81 min), RMSE 18.52 min (17.56–19.50 min), and MAPE 24.56% (23.50–25.66%) in the HA sample. In the KA sample, it achieved an MAE of 14.05 min (13.43–14.67 min), RMSE 19.24 min (18.19–20.34 min), and MAPE 22.96% (21.92–24.04%).

However, confidence intervals of XGBoost and linear multivariable regression overlapped in all cases (Table 2).

**Table 2:** Performance of the models on training data with cross-validation

Indication	Model	Mean absolute percentage error (%)	Root mean squared error (min)	Mean absolute error (min)
Hip (*n* = 1,578)	XGBoost	22.55 (21.60-23.52)	17.53 (16.64–18.46)	12.60 (12.10-13.11)
	Linear multivariable regression	24.56 (23.50-25.66)	18.52 (17.56–19.50)	13.26 (12.72–13.81)
Knee (*n* = 1,200)	XGBoost	20.94 (20.07–21.84)	18.04 (17.11-19.00)	13.21 (12.64–13.79)
	Linear multivariable regression	22.96 (21.92–24.04)	19.24 (18.19–20.34)	14.05 (13.43–14.67)

95% confidence intervals in parenthesis. Confidence intervals were derived using bootstrapping with 10,000 repetitions

#### Performance on test data (multi-hospital analysis)

Performance on test data revealed comparable performance of the models as on training data with CV.

For XGBoost, performance decreased slightly, with a MAE of 12.13 min (11.27–13.02 min), RMSE 17.18 min (15.42–19.00 min) and a MAPE of 22.66% (21.1-24.28%) in the HA sample, and a MAE of 13.61 min (12.56–14.73 min), RMSE of 19.03 min (16.93–21.64 min) and a MAPE of 23.30% (19.96 − 23.61%) in the KA sample.

In contrast, the performance of linear multivariable regression was slightly better on test than on training data, with a MAE of 12.51 min (11.63–13.44 min), RMSE of 17.68 min (15.94–19.50 min) and a MAPE of 23.89% (22.16–25.72%) in the HA sample, and a MAE of 13.55 min (12.45–14.69 min), RMSE of 19.24 min (17.04–21.64 min) and a MAPE of 21.51% (19.66 -23.49%) in the KA sample (Table 3).

**Table 3:** Performance of the models on test data

Indication	Model	Mean absolute percentage error (%)	Root mean squared error (min)	Mean absolute error (min)
Hip (*n* = 526)	XGBoost	22.66 (21.10-24.28)	17.18 (15.42-19.00)	12.13 (11.27–13.02)
	Linear multivariable regression	23.89 (22.16–25.72)	17.68 (15.94–19.50)	12.51 (11.63–13.44)
Knee (*n* = 400)	XGBoost	23.30 (19.96–23.61)	19.03 (16.93–21.39)	13.61 (12.56–14.73)
	Linear multivariable regression	21.51 (19.66–23.49)	19.24 (17.04–21.64)	13.55 (12.45–14.69)

95% confidence intervals in parenthesis. Confidence intervals were derived using bootstrapping with 10,000 repetitions

A) Hip arthroplasty

Similar to the training data set, XGBoost achieved better point estimates for performance, but confidence intervals of XGBoost and regression were overlapping.

#### Feature importance (multi-hospital analysis)

Feature importance for hip and knee arthroplasty revealed that the most important predictor was surgery performed in one specific hospital (“Hospital nr. 8”), with surgeries performed at this hospital resulting in reduced DOS. “Hospital nr. 3” also had a (smaller) negative impact on DOS.

Further, a higher number of performed surgeries by the leading physician, the involvement of a chief physician in the surgery, a lower number of specialist surgeons, a lower number of surgeons in training and a lower number of specialist surgeons, a lower BMI and a lower weight of the patient were associated with a lower DOS in HA patients.

**Fig. 3:**
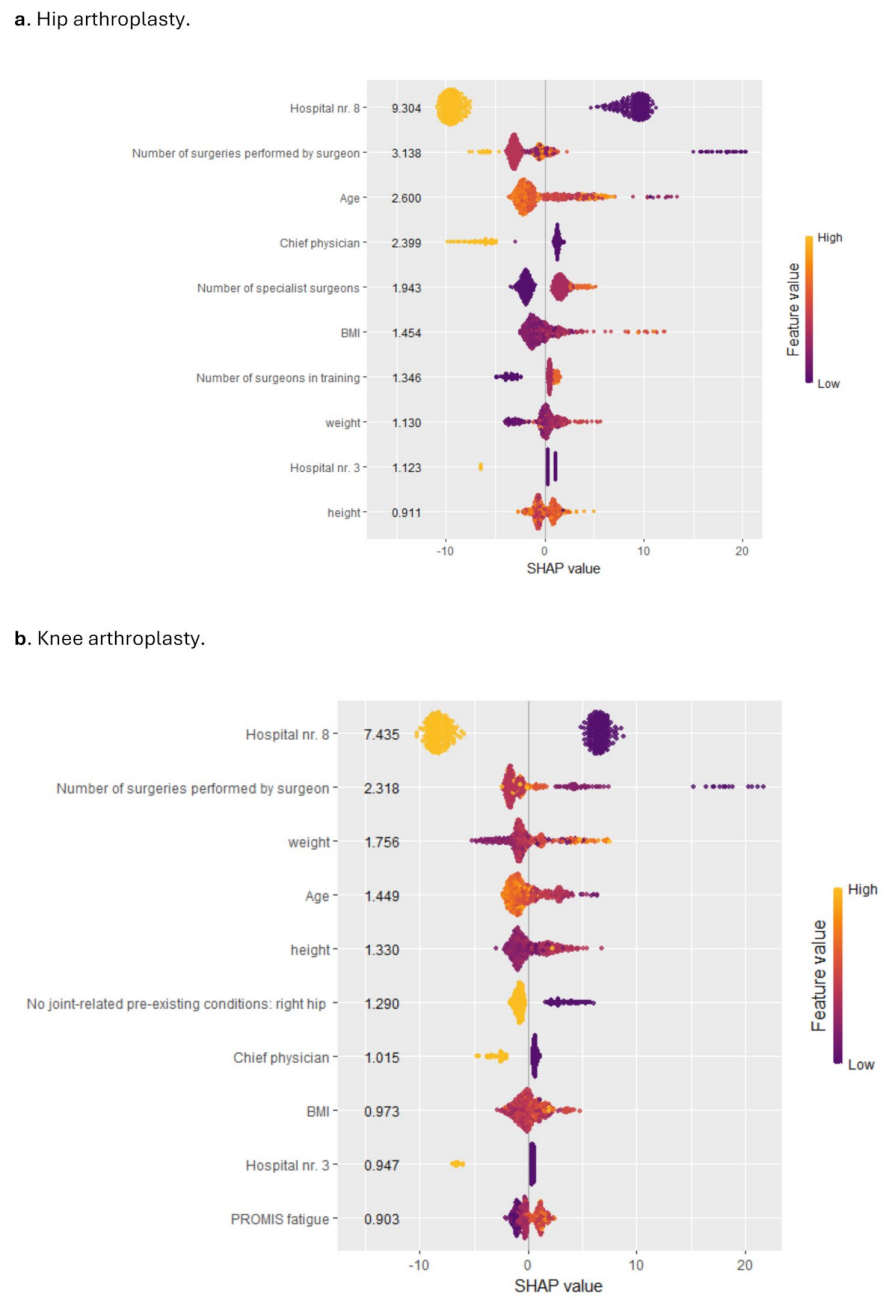
SHAP analysis of DOS for the KA and HA sample on the test dataset for the multi-hospital analysis

In KA patients, in addition, lower PROMIS fatigue scores and a lack of joint-related pre-existing conditions in the right hip were associated with lower DOS (Fig. 3).

### Single hospital deep dive

In practice, DOS prediction may be more relevant on the hospital-level than on the level of multiple hospitals. Therefore, we performed a deep-dive analysis of the same prediction task for the hospital (“Hospital nr. 8”) with the highest case-volumes in our dataset to demonstrate practicability of the potential operating room planning tool from a single-hospital perspective. In comparison to the multi-hospital setting, average DOS in the single-hospital dataset was lower, with 46.1 min (SD: 16.9) for HA patients and 52.6 min (SD: 15.3) for KA patients.

#### Performance on training data (single-hospital analysis)

On training data, the performance of all models was slightly better compared to the multi-hospital setting. For hip arthroplasty, the MAE for XGBoost was 1.44 min lower than for linear multiple regression (10.89 min vs. 12.33 min). Confidence intervals were not overlapping. For knee arthroplasty, the difference was even larger, with the MAE of XGBoost being 3.11 min lower than linear multiple regression (10.14 min vs. 13.25 min). Here, the difference was significant as confidence intervals were not overlapping (Table 4).

**Table 4:** Performance of the models on training data with cross-validation

Indication	Model	Mean absolute percentage error (%)	Root mean squared error (min)	Mean absolute error (min)
Hip (*n* = 810)	XGBoost	23.73 (22.28–25.20)	15.54 (14.26–16.81)	10.89 (10.25–11.55)
	Linear multivariable regression	40.40 (29.52–55.74)	17.32 (16.01–18.63)	12.33 (11.63–13.04)
Knee (*n* = 550)	XGBoost	19.07 (17.57–20.60)	14.92 (13.11–16.87)	10.14 (9.38–10.91)
	Linear multivariable regression	35.35 (27.64–45.30)	19.26 (17.42–21.14)	13.25 (12.30-14.25)

95% confidence intervals in parenthesis. Confidence intervals were derived using bootstrapping with 10,000 repetitions

#### Performance on test data (single-hospital analysis)

On unforeseen test data, performance of all models decreased. In the hip arthroplasty sample, XGBoost’s MAE was 10.87 min and therefore 0.26 min lower than that of linear multiple regression (MAE = 11.13 min). For knee arthroplasty, the difference between XGBoost and linear multivariable regression was 1.11 min, with the MAE of XGBoost at 12.53 min. Confidence intervals were overlapping both in the KA and the HA sample (Table 5).

**Table 5:** Performance of the models on unforeseen test data

Indication	Model	Mean absolute percentage error (%)	Root mean squared error (min)	Mean absolute error (min)
Hip (*n* = 271)	XGBoost	23.30 (20.89–25.92)	15.75 (13.53–18.02)	10.87 (9.76–12.05)
	Linear multivariable regression	24.59 (21.83–27.61)	16.00 (13.65–18.49)	11.13 (10.02–12.29)
Knee (*n* = 184)	XGBoost	21.04 (18.49–23.87)	17.91 (13.96–22.01)	12.53 (11.04–14.15)
	Linear multivariable regression	27.08 (23.33–31.22)	20.23 (16.06–24.4)	13.64 (11.89–15.52)

95% confidence intervals in parenthesis. Confidence intervals were derived using bootstrapping with 10,000 repetitions

a) Hip arthroplasty

#### Feature importance

Feature importance was mostly in line with the multi-hospital setting. As hospital dummies were not present in the single hospital analysis, PROMs and self-reported pain gained importance. The number of performed surgeries was the most important predictor in both samples.

In the HA sample, the number of performed surgeries by the physician, high age, high self-reported pain in either hip, high height or BMI, a specific PROMIS fatigue answer, high weight and the absence of a chief physician were associated with increased DOS. For EQ-5D-5 L scores, the pattern was less clear with a tendency of lower scores being associated with increased DOS.

**Fig. 4:**
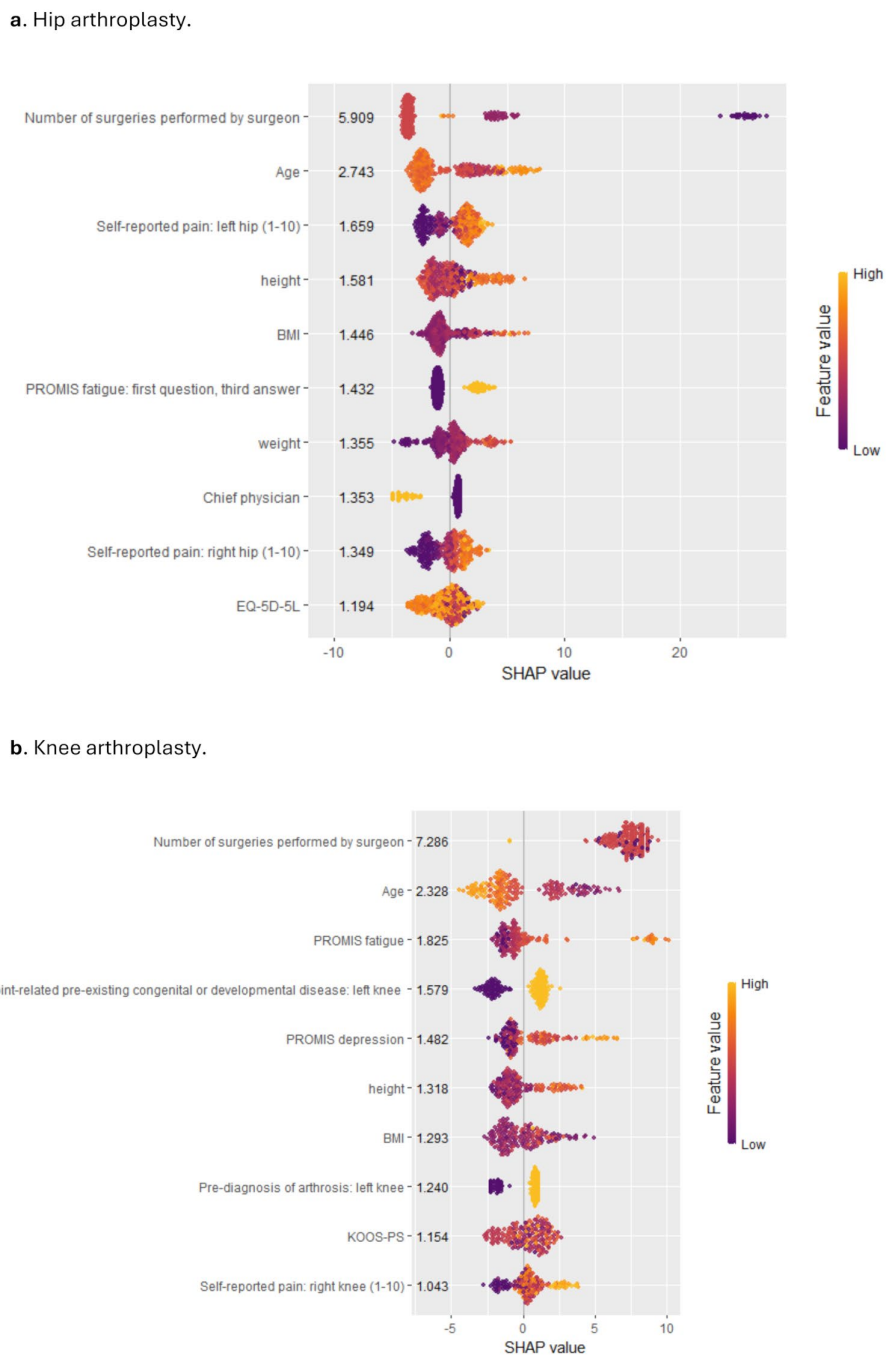
SHAP beewswarm plot of DOS for the KA and HA sample on the test dataset for the single hospital deep dive

In the KA sample, a high number of surgeries performed by the physician, high age, high PROMIS fatigue, pre-existing congenital or developmental diseases, high number of PROMIS depression, high height, arthrosis and high self-reported pain were associated with increased DOS. For KOOS-PS and BMI, a mixed pattern appeared (Fig. 4).

**Fig. 5:**
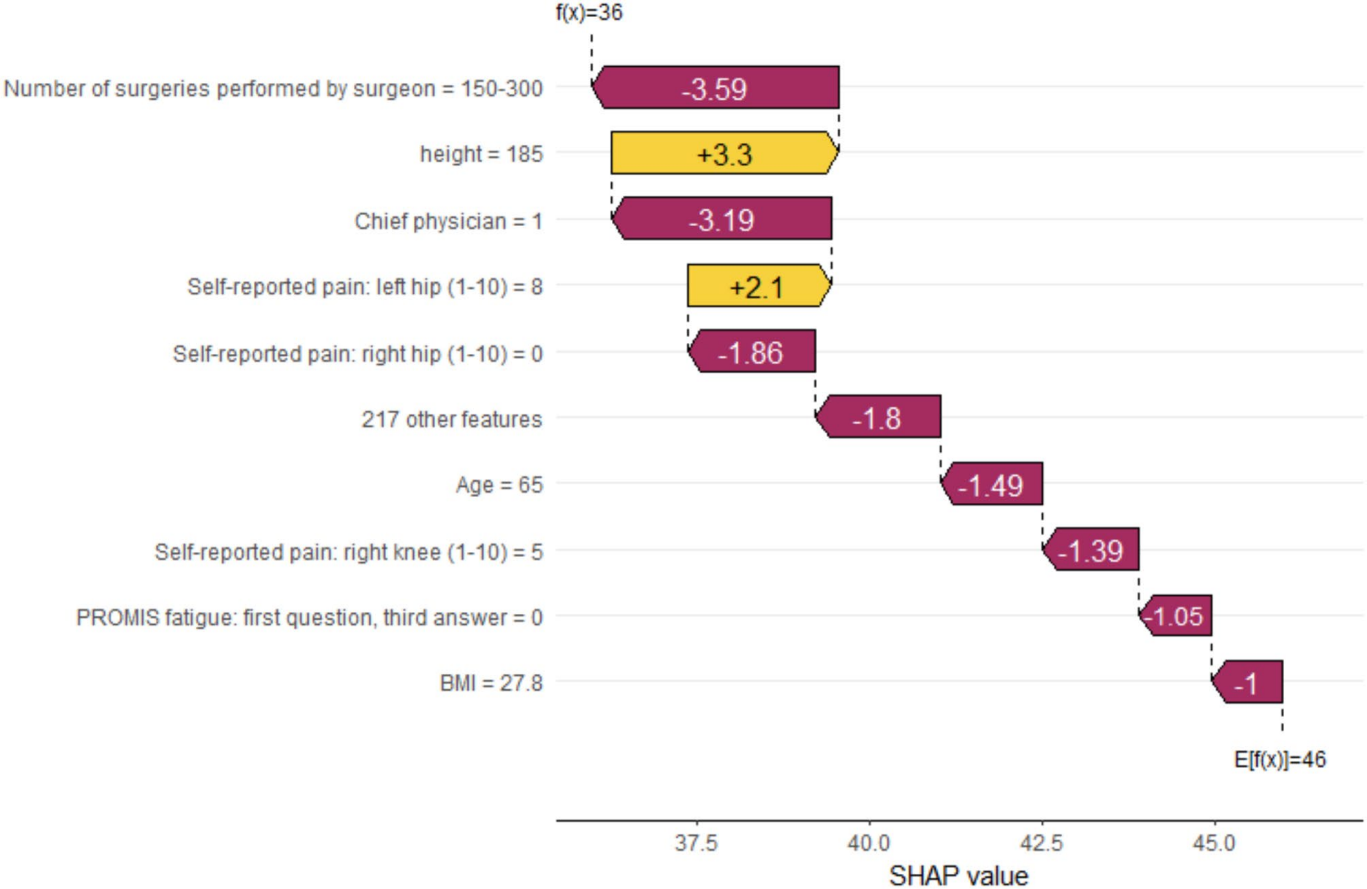
SHAP waterfall plot for a random patient (example) for the top 10 features. The plot must be read from bottom to top. The expected value of DOS was E[f(x)] = 46 (i.e. “blank” expected DOS without features). After adjusting for features, the expected DOS (= f(x)) was 36 for this patient (see top). Variables other than the top 10 are summarized as “217 other features”– which collectively decreased DOS by 1.8 min

In Fig. 5 we see a SHAP waterfall plot, which illustrates how the top 10 features influenced predicted DOS for an individual patient. We observe that the expected value before conditioning on certain features was 46 min (i.e. average DOS for HA patients in hospital 8). For this patient, the surgeon had an experience of 150–300 surgeries. Therefore, DOS decreased by 3.59 min. Among the top 10 predictors, only self-reported pain of the patient in the left hip, as well as the patient’s height increased predicted DOS. All other features decreased predicted DOS (Fig. 5).

## Discussion

We applied XGBoost, a state-of-the-art ML method, and linear multivariable regression as baseline comparison model [7] in order to predict DOS in HA and KA patients, both in a multi-hospital as well as a single-hospital setting. We found that DOS in the multi-hospital setting could be predicted with a MAE of 12.13 min for HA (XGBoost) and 13.55 min for KA patients (linear regression) on unforeseen test data. In the single-hospital setting, performance was better, with XGBoost as single-best model in predicting DOS with a MAE of 10.87 min for HA and 12.53 min for KA patients. MAPE values were 22.66% (HA) and 21.51% (KA) in the multi-hospital setting on test data, and 21.04% (KA) to 23.30% (HA) in the single hospital setting for the best models. Although XGBoost performed overall slightly favorable in comparison to linear regression, statistically significant differences could only be detected with respect to MAE in the single-hospital training dataset CV application (Table 4). We observed, overall, that models did not overfit notably on the training data.

Comparing our results to other studies, we found that the precision of our prediction models was in an acceptable range. One study predicting total procedure time (in contrast to our study, which predicted surgeon-controlled time) reported a MAE of 31.3 min in a large population (> 80,000) with various diagnosis and an average duration of 150 min [20]. Percentagewise, MAPE was about +/- 21%, a value comparable to our application, while MAE was substantially higher. Another study with heterogeneous procedures from Tel Aviv reported slightly better results regarding MAPE [32] compared to our application. However, given that their sample was much larger than ours, our MAPE appears to be low.

Different from previous classification studies [17–19], this is the first study in knee or hip arthroplasty patients that demonstrated reasonable performance on practically relevant metrics (MAE and MAPE) of high-dimensional prediction tools to forecast DOS as continuous outcome. We argue that our results are of higher practical relevance than binary prediction studies, as only continuous outcome predictions allow for accurate surgery planning. An accurate prediction and superior planning of OR capacity may allow for increased OR efficiency due to tighter OR scheduling for patients with low DOS (and therefore less idle time and increased throughput), as well as due to reduced waiting times or scheduling shifts in case of patients with high DOS. In practice, our prediction models may improve OR scheduling efficiency, and support targeted staffing (or, if staffing is fixed, patient scheduling can be adapted for given staffing). Model-based OR planning that incorporates various features and allows for complex interactions of variables in planning is more advanced than surgeon-based OR planning, for it was shown that surgeons systematically overestimate expected surgeon-controlled time [33]. Despite OR efficiency issues, accurately predicted DOS may also improve patient safety. Since long DOS is associated with increased risk of revision, readmission, complications, renal impairments and infections [1–6], patients with predicted long DOS can be treated in a more appropriate manner, e.g. through performance of surgery with more experienced surgeons / chief physicians, in order to proactively reduce DOS. Therefore, adverse event risk may be mitigated, and patient safety increased.

In line with previous studies [17–19, 34, 35], we found that important predictors of DOS were surgeons’ experience, age, BMI, weight, and height. However, in contrast to previous studies, we did not find that gender or comorbidities (i.e. Charlson categories) were top predictors. Furthermore, we found that baseline PROMs, which aim to measure constructs such as quality of life or function, may serve as relevant features. This finding underlines the value of routinely collecting PROMs in patient care as they may be of various practical relevance. In fact, we found that PROMs may be superior predictors of DOS compared to gender or Charlson comorbidities (however, PROMs may of course correlate with certain comorbidities). In addition, even after controlling for several peri-operative variables, we found that hospital dummies absorbing other unobserved hospital characteristics have important predictive power in the multi-hospital setting. This may be strongly influenced by the quality of the surgery department and the interaction between the hospital and other included variables. For example, specific patient types may experience different DOS in different hospitals due to better adaptation of a hospital to their needs. For example, previous evidence has found that operation room team characteristics are among the most important predictors of duration of surgery [35]. In the case of our study, certain operation team characteristics may have been even more important than the plain experience of the chief surgeon (see Fig. 3). This makes the case for further investigation of hospital-specific factors that are associated with DOS even after controlling for a broad set of patient-specific and surgery-team-specific variables, and potentially may provide insights into quality-differences of hospitals.

The multi-hospital analysis is further important because of the abovementioned aspects of risks for patients that are associated with increased DOS. Patients with high risk of high DOS may be allocated to hospitals with lower expected DOS to mitigate risks. Nevertheless, it should be noted that SHAP values do not identify causal effects of a given feature and the predicted output (i.e. DOS) [32, 36]. Therefore, results must be interpreted with caution. It could be, for example, that a high number of specialist surgeons is only associated with a higher DOS because of a special type of patient that is operated, and it may be not that the high number of specialist surgeons that *causes* the high DOS. Also, it may be that a higher number of specialists is related to practical training. Thus, further reasoning about the causes of a variable’s influence on predictions is necessary.

Finally, this study has limitations. First, the dataset was relatively small for a ML application, especially as we needed to exclude two hospitals from the dataset for erroneous DOS reporting. Therefore, larger datasets are necessary to identify whether predictive performance can be increased, and whether XGBoost may be able to outperform linear regression in larger applications. Second, the dataset included a large set of unique, not routinely available variables, especially PROMs. While a lot of these variables could be easily gathered through patient self-reporting, PROMs require typically a fee for use. Further, we did not include sickness fund data in our predictive models. Sickness fund data may be an additional source of valuable information and should be included in future works (if available). Third, we could not provide a practical experiment on whether predictive performance will actually improve OR efficiency and patient safety. Therefore, pilot projects may be initiated to test advanced analytics in OR planning for HA and KA patients. Fourth, we defined DOS as surgeon-controlled-time. Nonetheless, there may be parts of operation room planning that are before and after this period which we do not cover. Practical testing may be necessary to determine if surgeon-controlled time is the best metric for DOS prediction, or whether other measures, e.g. induction of anesthesia and time between surgeries, should be included. Fifth and finally, we could only identify association of predictors and DOS, but not causality. This may challenge practical implications on altering certain features to reduce DOS.

## Conclusion

This ML application study demonstrated that DOS can be predicted with practical relevance for OR efficiency optimization. XGBoost generally perform slightly better on point estimates but did not outperform traditional linear multivariable regression statistically significantly on unforeseen test data. Further research using larger datasets is required to improve performance. Practitioners may explore whether DOS defined as surgeon-controlled-time or another metric works best for OR planning optimization.

## Electronic supplementary material

Below is the link to the electronic supplementary material.

Supplementary Material 1

Supplementary Material 2

Supplementary Material 3

## Data Availability

The data associated with the paper are not publicly available due to legal restrictions.
