# Supplementary material for: Leveraging machine learning for duration of surgery prediction in knee and hip arthroplasty – a development and validation study
Source: BMC Med Inform Decis Mak. 2025 Mar 3;25:106. doi: 10.1186/s12911-025-02927-7 (PMC11877953; doi:10.1186/s12911-025-02927-7)
Supplement: Supplementary file 1 — Supplementary Material 1 [file 12911_2025_2927_MOESM1_ESM.rtf]

Supplementary File 1: Distribution of duration of surgery for hospitals 4 and 9.


Supplementary Figure 1: Duration of surgery for hip arthroplasty patients for hospital 4.

Supplementary Figure 2: Duration of surgery for knee arthroplasty patients for hospital 4.


Supplementary Figure 3: Duration of surgery for hip arthroplasty patients for hospital 9.


Supplementary Figure 4: Duration of surgery for knee arthroplasty patients for hospital 9.
