# Supplementary material for: Leveraging machine learning for duration of surgery prediction in knee and hip arthroplasty – a development and validation study
Source: BMC Med Inform Decis Mak. 2025 Mar 3;25:106. doi: 10.1186/s12911-025-02927-7 (PMC11877953; doi:10.1186/s12911-025-02927-7)
Supplement: Supplementary file 2 — Supplementary Material 2 [file 12911_2025_2927_MOESM2_ESM.docx]

**Table 1. Description of included variables.**

| **Variable** | **Description** |
| --- | --- |
| gender | Gender |
| I_trauma_HR | Joint related pre-existing conditions - trauma or ligament injury - hip r. - baseline hip/knee osteoarthritis (ICHOM) |
| I_trauma_HL | Joint related pre-existing conditions - trauma or ligament injury - hip left - baseline hip/knee osteoarthritis (ICHOM) |
| I_trauma_KR | Joint related pre-existing conditions - trauma or ligament injury - knee r. - baseline hip/knee osteoarthritis (ICHOM) |
| I_trauma_KL | Joint-related pre-existing conditions - trauma or ligament injury - left knee - baseline hip/knee osteoarthritis (ICHOM) |
| I_dev_HR | Joint-related pre-existing conditions - Congenital or developmental - Hip r. - Baseline hip/knee osteoarthritis (ICHOM) |
| I_dev_HL | Joint-related pre-existing conditions - Congenital or developmental disease - hip left - baseline hip/knee osteoarthritis (ICHOM) |
| I_dev_KR | Joint-related pre-existing conditions - Congenital or developmental disease - Knee r. - Baseline hip/knee osteoarthritis (ICHOM) |
| I_dev_KL | Joint-related pre-existing conditions - Congenital or developmental disease - Left knee - Baseline hip/knee osteoarthritis (ICHOM) |
| I_joint_HR | Joint-related pre-existing conditions - Other joint disease - Hip r. - Baseline hip/knee osteoarthritis (ICHOM) |
| I_joint_HL | Joint-related pre-existing conditions - Other joint diseases - Left hip - Baseline hip/knee osteoarthritis (ICHOM) |
| I_joint_KR | Joint-related pre-existing conditions - Other joint diseases - Knee r. - Baseline hip/knee osteoarthritis (ICHOM) |
| I_joint_KL | Joint-related pre-existing conditions - Other joint diseases - Left knee - Baseline hip/knee osteoarthritis (ICHOM) |
| I_no_HR | Joint-related pre-existing conditions - None - Hip r. - Baseline hip/knee osteoarthritis (ICHOM) |
| I_no_HL | Joint related pre-existing conditions - None - Hip left - Baseline hip/knee osteoarthritis (ICHOM) |
| I_no_KR | Joint-related pre-existing conditions - None - Knee r. - Baseline hip/knee osteoarthritis (ICHOM) |
| I_no_KL | Joint-related past medical history - None - Knee left - Baseline hip/knee osteoarthritis (ICHOM) |
| I_repl_HR | Joint-related surgical history - Joint replacement - Hip r. - Baseline hip/knee osteoarthritis (ICHOM) |
| I_repl_HL | Joint Surgical History - Joint Replacement - Left Hip - Baseline Hip/Knee Osteoarthritis (ICHOM) |
| I_repl_KR | Joint surgical history - joint replacement - knee right - baseline hip/knee osteoarthritis (ICHOM) |
| I_repl_KL | Joint surgical history - joint replacement - left knee - baseline hip/knee osteoarthritis (ICHOM) |
| I_osteotom_HR | Joint surgical history - osteotomy - right hip - baseline hip/knee osteoarthritis (ICHOM) |
| I_osteotom_HL | Joint surgical history - osteotomy - left hip - baseline hip/knee osteoarthritis (ICHOM) |
| I_osteotom_KR | Joint surgical history - osteotomy - knee r. - baseline hip/knee osteoarthritis (ICHOM) |
| I_osteotom_KL | Joint surgical history - osteotomy - left knee - baseline hip/knee osteoarthritis (ICHOM) |
| I_osteosyn_HR | Joint surgical history - osteosynthesis/surgical fracture repair - hip right - baseline hip/knee osteoarthritis (ICHOM) |
| I_osteosyn_HL | Joint surgical history - osteosynthesis/surgical fracture repair - hip left - baseline hip/knee osteoarthritis (ICHOM) |
| I_osteosyn_KR | Joint surgical history - osteosynthesis/surgical fracture repair - right knee - baseline hip/knee osteoarthritis (ICHOM) |
| I_osteosyn_KL | Joint surgical history - osteosynthesis/surgical fracture repair - left knee - baseline hip/knee osteoarthritis (ICHOM) |
| I_recon_HR | Joint surgical history - hip ligament reconstruction - right hip - baseline hip/knee osteoarthritis (ICHOM) |
| I_recon_HL | Joint surgical history - hip ligament reconstruction - left hip - baseline hip/knee osteoarthritis (ICHOM) |
| I_recon_KR | Joint surgical history - knee ligament reconstruction - right knee - baseline hip/knee osteoarthritis (ICHOM) |
| I_recon_KL | Joint Surgical History - Knee Ligament Reconstruction - Knee Left - Baseline Hip/Knee Osteoarthritis (ICHOM) |
| I_arth_HR | Joint Surgical History - Other Arthroscopic Procedures - Hip Right - Baseline Hip/Knee Osteoarthritis (ICHOM) |
| I_arth_HL | Joint Surgical History - Other Arthroscopic Procedures - Left Hip - Baseline Hip/Knee Osteoarthritis (ICHOM) |
| I_arth_KR | Joint Surgical History - Other Arthroscopic Procedures - Knee Right - Baseline Hip/Knee Osteoarthritis (ICHOM) |
| I_arth_KL | Joint Surgical History - Other Arthroscopic Procedures - Left Knee - Baseline Hip/Knee Osteoarthritis (ICHOM) |
| I_chirno_HR | Joint related surgical history - None - Hip r. - Baseline hip/knee osteoarthritis (ICHOM) |
| I_chirno_HL | Joint surgical history - None - Hip left - Baseline hip/knee osteoarthritis (ICHOM) |
| I_chirno_KR | Joint surgical history - None - Knee right - Baseline hip/knee osteoarthritis (ICHOM) |
| I_chirno_KL | Joint Surgical History - None - Knee Left - Baseline Hip/Knee Osteoarthritis (ICHOM) |
| height | Body height (in cm) - History of knee-hip osteoarthritis (PROMoting Quality) |
| weight | Body weight (in kg) - anamnesis knee- hip arthrosis (PROMoting Quality) |
| PQ_arth_HR | Pre-diagnosis of arthrosis - Right hip - Anamnesis knee- hip arthrosis (PROMoting Quality) |
| PQ_arth_HL | Pre-diagnosis of arthrosis - Left hip - Anamnesis knee- hip arthrosis (PROMoting Quality) |
| PQ_arth_KR | Pre-diagnosis of arthrosis - Right knee - Anamnesis knee- hip arthrosis (PROMoting Quality) |
| PQ_arth_KL | Pre-diagnosis of arthrosis - Left knee - anamnesis knee- hip arthrosis (PROMoting Quality) |
| PQ_arthno | Pre-diagnosis of arthrosis - No - anamnesis knee- hip arthrosis (PROMoting Quality) |
| PQ_OParth_HR | Operations for therapy of an arthrosis in the past year - Right hip - Anamnesis knee- hip arthrosis (PROMoting Quality) |
| PQ_OParth_HL | Operations for the therapy of an arthrosis in the past year - Left hip - anamnesis knee- hip arthrosis (PROMoting Quality) |
| PQ_OParth_KR | Operations for the therapy of an arthrosis in the past year - Right knee - anamnesis knee- hip arthrosis (PROMoting Quality) |
| PQ_OParth_KL | Operations for the therapy of an arthrosis in the past year - Left knee - anamnesis knee- hip arthrosis (PROMoting Quality) |
| PQ_OParthno | Operations for therapy of an arthrosis in the past year - None - Anamnesis knee- hip arthrosis (PROMoting Quality) |
| PQ_therarth1 | Therapies for osteoarthritis-related hip/knee problems in the past year - Information/advice - History of knee- hip osteoarthritis (PROMoting Quality) |
| PQ_therarth2 | Therapies for osteoarthritis-related hip/knee problems in the past year - Self-administered care - Anamnesis knee- hip osteoarthritis (PROMoting Quality) |
| PQ_therarth3 | Therapies for osteoarthritis-related hip/knee problems in the past year - Non-surgical clinical care - History of knee- hip osteoarthritis (PROMoting Quality) |
| PQ_therarth4 | Therapies for osteoarthritis-related hip/knee problems in the past year - Operations - Anamnesis knee- hip osteoarthritis (PROMoting Quality) |
| PQ_therarthno | Therapies for osteoarthritis-related hip/knee problems in the past year - None - Anamnesis knee- hip osteoarthritis (PROMoting Quality) |
| PQ_care_selfhelp | Visited providers for therapy osteoarthritis-related hip/knee problems in past year - Health educator/support group - History of knee- hip osteoarthritis (PROMoting Quality) |
| PQ_care_gpnutphy | Visited providers for therapy osteoarthritis-related hip/knee problems in past year - Nutritionist/physiotherapist/family physician - History of knee- hip osteoarthritis (PROMoting Quality) |
| PQ_care_rheu | Visited providers for therapy of osteoarthritis-related hip/knee problems in the past year - Rheumatologist - History of knee/hip osteoarthritis (PROMoting Quality) |
| PQ_care_ortho | Visited providers for therapy of osteoarthritis-related hip/knee problems in the past year - Orthopedic surgeon - History of knee/hip osteoarthritis (PROMoting Quality) |
| PQ_care_healer | Visited providers for therapy of osteoarthritis-related hip/knee problems in the past year - Orthopedic practitioner - Anamnesis knee- hip osteoarthritis (PROMoting Quality) |
| PQ_careno | Visited providers for therapy of osteoarthritis-related hip/knee problems in the past year - None - Medical history of knee/hip osteoarthritis (PROMoting Quality) |
| smoker | Smoker - History of knee/hip osteoarthritis (PROMoting Quality) |
| EQ_health | EQ-VAS score |
| EQ_total | EQ-5D-5L score |
| PROMIS_dep | Score: Depressiveness - PROMIS Depressiveness & Exhaustion |
| PROMIS_fat | Score: Exhaustion - PROMIS Depressiveness & Exhaustion |
| HOOS_KOOS | HOOS-PS / KOOS-PS Score |
| PQ_pain_HR | Average pain intensity right hip within the last 7 days (1-10) |
| PQ_pain_HL | Average pain intensity left hip within the last 7 days (1-10) |
| PQ_pain_KR | Average pain intensity right knee within the last 7 days (1-10) |
| PQ_pain_KL | Average pain intensity left knee within the last 7 days (1-10) |
| PQ_back | Average pain intensity back within the last 7 days (1-10) |
| bmi | Body Mass index |
| age_at_surgery | Age at the time of surgery |
| caredegree_high | Having a caredegree according to the German "Pflegegrad" classification |
| active | At least one hour of physical activity per week |
| worker | At least part time worker |
| HOOS_1_2 | HOOS-PS, subdimension 1, second answer |
| HOOS_1_3 | HOOS-PS, subdimension 1, third answer |
| HOOS_1_4 | HOOS-PS, subdimension 1, fourth answer |
| HOOS_1_5 | HOOS-PS, subdimension 1, fifth answer |
| HOOS_2_2 | HOOS-PS, subdimension 2, second answer |
| HOOS_2_3 | HOOS-PS, subdimension 2, third answer |
| HOOS_2_4 | HOOS-PS, subdimension 2, fourth answer |
| HOOS_2_5 | HOOS-PS, subdimension 2, fifth answer |
| HOOS_3_2 | HOOS-PS, subdimension 3, second answer |
| HOOS_3_3 | HOOS-PS, subdimension 3, third answer |
| HOOS_3_4 | HOOS-PS, subdimension 3, fourth answer |
| HOOS_3_5 | HOOS-PS, subdimension 3, fifth answer |
| HOOS_4_2 | HOOS-PS, subdimension 4, second answer |
| HOOS_4_3 | HOOS-PS, subdimension 4, third answer |
| HOOS_4_4 | HOOS-PS, subdimension 4, fourth answer |
| HOOS_4_5 | HOOS-PS, subdimension 4, fifth answer |
| HOOS_5_2 | HOOS-PS, subdimension 5, second answer |
| HOOS_5_3 | HOOS-PS, subdimension 5, third answer |
| HOOS_5_4 | HOOS-PS, subdimension 5, fourth answer |
| HOOS_5_5 | HOOS-PS, subdimension 5, fifth answer |
| PROMIS_fat1_2 | Promis Fatique Dimension 1: I felt exhausted within the last 7 days: A bit |
| PROMIS_fat1_3 | Promis Fatique Dimension 1: I felt exhausted within the last 7 days: Medium |
| PROMIS_fat1_4 | Promis Fatique Dimension 1: I felt exhausted within the last 7 days: Not at all |
| PROMIS_fat1_5 | Promis Fatique Dimension 1: I felt exhausted within the last 7 days: Very |
| PROMIS_fat2_2 | Promis Fatique Dimension 2: I felt exhausted within the last 7 days: Not at all |
| PROMIS_fat2_3 | Promis Fatique Dimension 2: I felt exhausted within the last 7 days: A bit |
| PROMIS_fat2_4 | Promis Fatique Dimension 2: I felt exhausted within the last 7 days: Not at all |
| PROMIS_fat2_5 | Promis Fatique Dimension 2: I felt exhausted within the last 7 days: Not at all |
| PROMIS_fat3_2 | Promis Fatique Dimension 2: I felt drained in general within the last 7 days: Not at all |
| PROMIS_fat3_3 | Promis Fatique Dimension 2: I felt drained in general within the last 7 days: A bit |
| PROMIS_fat3_4 | Promis Fatique Dimension 2: I felt drained in general within the last 7 days: A lot |
| PROMIS_fat3_5 | Promis Fatique Dimension 2: I felt drained in general within the last 7 days: Very |
| PROMIS_fat4_2 | Promis Fatique Dimension 2: I felt exhausted in general within the last 7 days: Not at all |
| PROMIS_fat4_3 | Promis Fatique Dimension 2: I felt exhausted in general within the last 7 days: A bit |
| PROMIS_fat4_4 | Promis Fatique Dimension 2: I felt exhausted in general within the last 7 days: Very |
| PROMIS_fat4_5 | Promis Fatique Dimension 2: I felt exhausted in general within the last 7 days: A lot |
| PROMIS_dep1_2 | PROMIS Depression , subdimension 1, second answer |
| PROMIS_dep1_3 | PROMIS Depression , subdimension 1, third answer |
| PROMIS_dep1_4 | PROMIS Depression , subdimension 1, fourth answer |
| PROMIS_dep1_5 | PROMIS Depression , subdimension 1, fifth answer |
| PROMIS_dep2_2 | PROMIS Depression , subdimension 2, second answer |
| PROMIS_dep2_3 | PROMIS Depression , subdimension 2, third answer |
| PROMIS_dep2_4 | PROMIS Depression , subdimension 2, fourth answer |
| PROMIS_dep2_5 | PROMIS Depression , subdimension 2, fifth answer |
| PROMIS_dep3_2 | PROMIS Depression , subdimension 3, second answer |
| PROMIS_dep3_3 | PROMIS Depression , subdimension 3, third answer |
| PROMIS_dep3_4 | PROMIS Depression , subdimension 3, fourth answer |
| PROMIS_dep3_5 | PROMIS Depression , subdimension 3, fifth answer |
| PROMIS_dep4_2 | PROMIS Depression , subdimension 4, second answer |
| PROMIS_dep4_3 | PROMIS Depression , subdimension 4, third answer |
| PROMIS_dep4_4 | PROMIS Depression , subdimension 4, fourth answer |
| PROMIS_dep4_5 | PROMIS Depression, subdimension 5, answer 1 |
| EQ_anxiety_2 | EQ anxiety: I'm not scared or depressed |
| EQ_anxiety_3 | EQ anxiety: I'm a bit scared or depressed |
| EQ_anxiety_4 | EQ anxiety: I'm medium scared or depressed |
| EQ_anxiety_5 | EQ anxiety: I'm heavily scared or depressed |
| EQ_pain_2 | EQ pain: I have medium pain or complains |
| EQ_pain_3 | EQ pain: I have no pain or complains |
| EQ_pain_4 | EQ pain: I have no pain or complains |
| EQ_pain_5 | EQ pain: I have small pain or complains |
| EQ_dailyactiv_2 | EQ daily activity: I have small problems doing my daily activities |
| EQ_dailyactiv_3 | EQ daily activity: I have huge problems doing my daily activities |
| EQ_dailyactiv_4 | EQ daily activity: I have no problems doing my daily activities |
| EQ_dailyactiv_5 | EQ daily activity: I am not able to do my daily activities |
| EQ_selfcare_2 | EQ selfcare: I do not have problems with cleaning myself or to put clothes on |
| EQ_selfcare_3 | EQ selfcare: I am not able to clean myself or to put clothes on |
| EQ_selfcare_4 | EQ selfcare: I have huge problems with cleaning myself or to put clothes on |
| EQ_selfcare_5 | EQ selfcare: I have medium-size problems with cleaning myself or to put clothes on |
| EQ_mobility_2 | EQ mobility: I have small problems with walking |
| EQ_mobility_3 | EQ mobility: I have medium problems with walking |
| EQ_mobility_4 | EQ mobility: I have no problems with walking |
| EQ_mobility_5 | EQ mobility: I am not able to walk |
| caredegree_2 | German care degree: Level 1 |
| caredegree_3 | German care degree: Level 2 |
| caredegree_4 | German care degree: Level 3 |
| caredegree_5 | German care degree: Level 4 |
| caredegree_6 | German care degree: Level 5 |
| duration_weeklyactiv_2 | Duration of weekly physical activity: More than 2 hours |
| duration_weeklyactiv_3 | Duration of weekly physical activity: About an hour |
| duration_weeklyactiv_4 | Duration of weekly physical activity: Zero |
| duration_weeklyactiv_5 | Duration of weekly physical activity: 30 minutes |
| jobeffort_2 | Phyisical activity during work: Can not judge |
| jobeffort_3 | Phyisical activity during work: Heavy |
| jobeffort_4 | Phyisical activity during work: Easy |
| jobeffort_5 | Phyisical activity during work: Medium |
| job_2 | Job: Not working (Student, Housewife/man) |
| job_3 | Job: Full-time |
| job_4 | Job: Part-time |
| job_5 | Job: Not able to work due to a disease other than arthrosis |
| job_6 | Job: Searching for work (able to work but currently jobless) |
| livsituation_2 | Living situation: Living alone |
| livsituation_3 | Living situation: Other |
| livsituation_4 | Living situation: Nursing home or hospital |
| education_1 | Finished at least high school |
| hospital_2 | Hospital Nr. 2 |
| hospital_3 | Hospital Nr. 3 |
| hospital_4 | Hospital Nr. 4 |
| hospital_5 | Hospital Nr. 5 |
| hospital_6 | Hospital Nr. 6 |
| hospital_7 | Hospital Nr. 7 |
| hospital_8 | Hospital Nr. 8 |
| hospital_9 | Hospital Nr. 9 |
| hospital_1 | Hospital Nr. 1 |
